# Increasing early infant male circumcision uptake in Zambia: Like father like son

**DOI:** 10.1371/journal.pone.0289819

**Published:** 2023-08-10

**Authors:** Stephen M. Weiss, Violeta J. Rodriguez, Ryan R. Cook, Kasonde Bowa, Robert Zulu, Oliver Mweemba, Royd Kamboyi, Jose Castro, Victoria Orrego Dunleavy, Maria L. Alcaide, Deborah L. Jones

**Affiliations:** 1 Department of Psychiatry and Behavioral Sciences, University of Miami Miller School of Medicine, Miami, Florida, United States of America; 2 Department of Psychology, University of Georgia, Athens, Georgia, United States of America; 3 Medicine, General Internal Medicine, and Geriatrics, Oregon Health & Science University, Portland, Oregon, United States of America; 4 University of Lusaka School of Medicine, Lusaka, Zambia; 5 University of Zambia School of Medicine, Lusaka, Zambia; 6 Department of Health Promotion and Education, School of Public Health, University of Zambia, Lusaka, Zambia; 7 Ministry of Health, Lusaka, Zambia; 8 Division of Infectious Diseases, Dept. of Medicine, University of Miami Miller School of Medicine, Miami, Florida, United States of America; 9 School of Communication, University of Miami, Miami, Florida, United States of America; University of Zimbabwe Faculty of Medicine: University of Zimbabwe College of Health Sciences, ZIMBABWE

## Abstract

Voluntary Medical Male Circumcision (VMMC) is an effective strategy for HIV prevention in areas with high prevalence of, and risk for, HIV. More than 361,000 male neonates are born each year in Zambia, many of whom could be eligible for Early-Infant Medical Circumcision (EIMC). Building on successful implementation strategies utilized in our Spear & Shield program, this pilot study, “Like Father, Like Son” (LFLS), evaluated the feasibility and acceptability of offering combined EIMC and VMMC services and couple-level behavioral interventions. A total of *N* = 702 pregnant women and their male partners (*n* = 351 couples) were recruited and enrolled. Couples were assessed twice pre-birth, 2 weeks post birth, and 6 months post birth. Expectant mothers were an average of 15.05 weeks pregnant (*SD* = 8.83). Thirty-nine pregnancies did not result in a live birth (11%), 14 couples withdrew from the study or were lost to follow-up prior to delivery (4%), and 148 babies were born female (42%), leaving 150 couples with a male infant in the analytic sample (43%). The LFLS study achieved significantly higher EIMC rates (35%) in comparison with previously observed EIMC study rates in Zambia (11%), and significantly higher than hypothetical comparison rates up to 30%. Relative to baseline rates, odds of VMMC among couples’ older sons increased by 31% at post-intervention and by 90% at two-weeks following birth. Overall, this pilot study found the LFLS intervention to be feasible, acceptable, and effective in doubling the rate of EIMC in comparison with a previous longitudinal study in Zambia. Future research should consider a family-centric approach to promotion of male circumcision for infants and adolescents. LFLS may be effective in promoting father-son “bonding” by MC status; a bond that may be a bridge to increase both EIMC and VMMC uptake in newborns and couples’ older sons and is a novel leverage point for promotion of this HIV prevention strategy.

## Introduction

Voluntary Medical Male Circumcision (VMMC) is an effective strategy for HIV prevention in areas with high HIV prevalence [1–3] and thus, high risk for HIV transmission. It is estimated that VMMC has the potential to prevent 3.4 million HIV infections in Africa across a 10-year span. Several large-scale studies have confirmed significant reductions of HIV acquisition by more than 60% in Sub-Saharan Africa [2–8]. The World Health Organization (WHO), Center for Disease Control and Prevention (CDC), and The President’s Emergency Plan for AIDS Relief (PEPFAR) have expressed strong support for VMMC programs in Sub-Saharan Africa, making demand generation programs essential to achieve national goals [9]. With 1.2 million people with HIV (PWH), a prevalence of 11.3%, and an incidence of almost 70,000 new cases annually, the Zambian context is an appropriate setting to implement VMMC uptake interventions as a means of curtailing the HIV/AIDS epidemic [10].

Upwards of 360,000 of male neonates eligible for Early-Infant Medical Circumcision (EIMC) are born each year in Zambia. The timing for male circumcision is crucial, and integration of circumcision during the neonatal period was proposed by the Government of Zambia Ministry of Health (GZMOH) in 2008 due to both practical and medical considerations [11]. Early-Infant Medical Circumcision occurs before boys become sexually active, and is safer, less expensive, and has shorter healing times compared to VMMC at older ages [12–14]. In addition to protection from future HIV infection (91%), EIMC carries additional benefits: reduced urinary tract infections (UTIs) (90%), and certain sexually transmitted infections (77%), and overall hygiene improvement (82%) [15]. However, increasing acceptability and uptake of EIMC must begin with education and engagement of Zambian parents on the benefits of this program.

Approximately 60% of expecting Zambian parents expressed concerns regarding EIMC, such as fragility of the neonate penis, quality of the EIMC, and post-procedural care [16]. And while there may be interest in undergoing EIMC, the number of EIMCs occurring has remained low. One study reported that among 2000 Zambian pregnant women provided with information on EIMC, 97% agreed they would circumcise their infant if they had a boy, but only 11% actually did so [17]. Similarly, the Zambia Operational Plan for EIMC/VMMC 2016–2020 reported significantly lower uptake of infant MC than originally targeted [18]. To help bridge the gap between contemplation and action, evidence-based interventions are necessary to increase MC uptake in reluctant parents. This study tested a biobehavioral demand generation strategy, providing on-site availability of EIMC and VMMC services embedded in the Spear & Shield (S&S) behavioral intervention. The S&S intervention significantly influenced the uptake of VMMC for men in Lusaka, Zambia, with participating individuals more than twice as likely to undergo VMMC compared to those in the control condition. Spear & Shield was then disseminated to 96 community health centers (CHCs), with relatively high rates of sustainability after four years [4, 19, 20].

This pilot study, entitled “Like Father, Like Son” (LFLS), evaluated the feasibility and acceptability of offering combined EIMC and VMMC services and behavioral interventions, building on successful implementation strategies utilized in S&S. It was theorized that the LFLS program could be a viable strategy to increase acceptability and uptake of EIMC among hesitant expecting parents. This study sought to determine whether father-son “bonding” by MC status would increase both VMMC and EIMC uptake in newborns, couples’ older sons, and fathers compared to historical EIMC and VMMC rates in Zambia.

## Methods

### Ethical review

Prior to study initiation, ethical approval was obtained from the University of Miami Institutional Review Board, the University of Zambia Biomedical Research Ethics Committee, and the Zambian National Health Research Authority. All procedures were followed in accordance with the ethical standards of the responsible committee on human experimentation (institutional and national) following the Helsinki Declaration of 1975, as revised in 2000. All participants provided written informed consent to participate in the study at enrollment. The LFLS protocol was registered on clinicaltrials.gov under trial number NCT04119414.

### Participants and procedures

**Fig 1:**
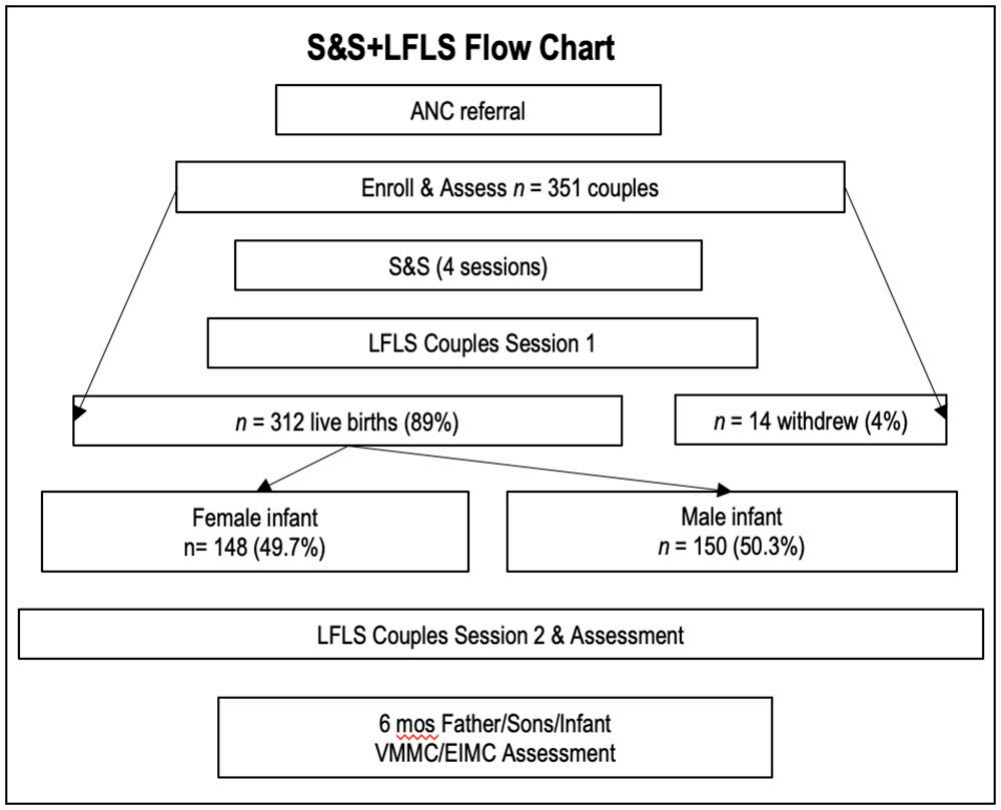
It describes the study timeline.

A total of *N* = 702 pregnant women and their male partners (*n* = 351 couples) were recruited and enrolled in Zambia from June 2019 to June 2022. Expecting mothers were an average of 15.05 weeks pregnant (*SD* = 8.83). Thirty-nine pregnancies did not end in a live birth (11%), 14 couples withdrew from the study or were lost to follow-up prior to delivery (4%), and 148 babies were born female (42%), leaving 150 couples with a male infant in the analytic sample (43%). The study timeline is described in Fig 1. Couples were assessed twice pre-birth, 2 weeks post birth, and 6 months post birth. Following 2 weeks post birth, participants were assessed regarding EIMC, VMMC, and plans to undergo EIMC and VMMC. During their last assessment (6 months post birth), participants were assessed regarding the feasibility and acceptability of the LFLS intervention.

Following screening and consent, a baseline audio computer-assisted self-interview (ACASI) assessment was conducted in English, Nyanja, or Bemba (primary local languages) in private study offices. Study staff demonstrated how to use the ACASI system and were available to assist participants and respond to queries regarding the assessment.

### Setting

The LFLS study was conducted in four urban community health centers (CHCs) in Lusaka District, Zambia. CHCs were selected based on catchment area ≥30,000; 2 healthcare providers for EIMC training and provision, 2 Voluntary Counseling and Testing (VCT) counselors and Antenatal care (ANC) staff available to offer an intervention, ≥150 births per month, a maternity ward, and dedicated space for both MC (EIMC and VMMC) procedures.

### Measures

#### Demographic characteristics

Participants’ demographic characteristics assessed included age, employment, religious affiliation, personal yearly income, education, marital status, pregnancy status, and whether they had their own children or were raising others’ children. Participants also provided information on both parents’ HIV status.

### Intervention

#### S&S + LFLS intervention training

Sites received S&S+LFLS training. The CHCs selected 2 VCT/ANC counselors/staff (or equivalent) to receive training to conduct the S&S+LFLS intervention. S&S training commenced with a 2 day intensive review of the S&S program manual followed by supervised “on the job” training sessions with a S&S staff trainer. The trainees participated in three 4 session S&S groups and five 2 session LFLS couple interventions. The first S&S/LFLS group and couple sessions were led by a study staff member with trainee(s) observing; second and third group sessions were co-led by trainee and study staff; fourth S&S session were led by trainee, with study staff observing. A similar pattern took place with the couple sessions. Following completion of the S&S+LFLS training, study staff reviewed trainee’s performance for both interventions and recommended certification, or additional training on either or both interventions. As EIMC decision making has been linked to mother’s HIV status, the LFLS couples component included elements from the couples’ voluntary HIV testing and counseling (CVCT), which applies a solution-focused model to avoid blame and instead centers on present and future behavior based on the CVCT Curriculum [21, 22]. The model has been endorsed by the GZMOH and integrated into ANC services’ standard of care. In Lusaka, HIV testing of pregnant women’s partners has increased from 2% in 2009 to 24.8% in 2015 [23]. Recent research in Zambia has demonstrated the high impact and cost-effectiveness of CVCT for HIV prevention [24], its reduction of unprotected sex within discordant couples [25], and the importance of influential networks in promoting CVCT [26]. The use of this model complemented the Zambian guidelines for PMTCT regarding promotion and support of male involvement in ANC [27], and provided a platform for the promotion of EIMC. Acceptability and feasibility for the program were also evaluated by its uptake.

#### S&S VMMC intervention theoretical model

The S&S VMMC intervention applied the Information Motivation Behavioral Skills (IMB) model to promote VMMC, providing VMMC related-information, increasing motivation to undergo VMMC, and promoting VMMC uptake and skills to reduce HIV risk and improve hygiene [28]. VMMC information included, realistic expectations, healing duration, sexual performance, abstaining from sex during healing, HIV/STI prevention, and women’s health issues. The utilization of information enhanced motivation for VMMC, which assists in the improvement of acceptability and attitudes, health behaviors, e.g., VMMC uptake, and increased use of sexual protection. The IMB model has been used with populations with HIV and limited literacy in a variety of settings [28–31].

#### LFLS EIMC intervention theoretical model

The LFLS EIMC intervention for parents also applied the Information Motivation Behavioral Skills (IMB) model to promote EIMC (EIMC related-information, increasing motivation to choose EIMC, taking up EIMC to reduce HIV/STD/UTI risk, and improve penile hygiene) [28]. The intervention provided EIMC Information (realistic expectations, healing duration, HIV/STD prevention, improved hygiene) that enhanced motivation for EIMC (improving acceptability & EIMC attitudes, improved hygiene, and healing) and health behavior (EIMC uptake).

#### Theory of reasoned action

The proposed EIMC pilot study was also guided by the theories of reasoned action (intentions influence attitudes and subjective norms, i.e., perceptions of social norms, motivation to comply, which influence beliefs about behavior [32] and planned behavior (perceived behavioral control influences intentions and behavior [33]) as predictors of EIMC [34]. Within this model, it was hypothesized that EIMC intentions and EIMC-related knowledge would result in higher EIMC uptake.

#### Model of EIMC decision making

The EIMC intervention incorporated factors associated with couples’ decision making [35] and leveraged the current MOH promotion of male engagement during pregnancy to increase EIMC uptake [18]. Potential factors and influential others (family members and peers who are decision makers, e.g., in-laws, aunts, uncles, grandfathers, brothers and sisters in law, friends) contributing to EIMC decision making include the father’s circumcision status, paternal grandparents’ circumcision attitudes, peer circumcision attitudes, father’s HIV status, mother’s HIV status, and circumcision attitudes. In session 2, couples were invited to bring an influential other to be included in the EIMC decision-making process.

#### S&S intervention

The S&S program was developed and culturally tailored for men and women from qualitative data on VMMC attitudes, preferences, and beliefs. This comprehensive risk reduction intervention consisted of 4 weekly 90-minute manualized group sessions. Female partners of participants were invited to participate in 4 comparable sessions on women’s VMMC-related issues [36]. The S&S intervention has been previously described [36]. Sessions addressed HIV/STDs, safer sex, male condoms, male circumcision, and sexual communication, and strategies for protecting against HIV, including VMMC. Sessions addressed VMMC as a permanent method of risk reduction and provided a forum for discussion of the VMMC procedure, concerns, limitations, and beliefs. Cognitive behavioral (CB) skill training heightened participants’ awareness of reactions to VMMC, reframing thoughts that impede VMMC uptake, condom use, and sexual communication. Potential benefits of VMMC for female partners were also addressed, and sessions included sharing experiences, concerns and attitudes about female condoms, perceptions of partners’ reactions to VMMC, role plays for problem solving and skill building. A peer who has undergone VMMC shared experiences, abstaining from sex during healing [37], and sexual satisfaction [38, 39], and answered questions about VMMC. The final session included a presentation on the VMMC procedure by a CHC VMMC provider, who discussed benefits and risks, post-VMMC recovery and resumption of sexual activities. High risk sexual behavior and alcohol/drugs were discussed, and communication skills were reviewed.

#### LFLS intervention

The existing standard of care [18] outlines the need to ensure that parents and guardians need time to understand the importance of EIMC before bringing babies in for EIMC and should be sensitized before delivery. ANC and VCT staff were encouraged to sensitize ANC attendee mothers and men undergoing VMMC about EIMC, and fliers for family members have been developed. Sensitized pregnant mothers and male partners with pregnant female partners who opted for enrolment were invited to participate along with their spouses. The LFLS program was developed collaboratively by the US and Zambia teams, and was culturally tailored for individual couples’ sessions using qualitative data from FGDs. The S&S manual guided the first phase of the comprehensive HIV risk reduction program (i.e., four 90-minute weekly group sessions, audio recorded). After the S&S sessions, each pregnant couple attended 2 private 90-minute LFLS sessions co-led by the male and female S&S facilitators, one couples session pre-delivery and one couples session immediately post-partum. Both sessions explored issues relevant to parental decision making related to VMMC and EIMC. The gap between session 1 (antenatal) and 2 (postpartum) provided opportunity for parents to consult with influential others.

The two LFLS sessions were based upon the community input, and “fine-tuned” using feasibility pilot data. To enhance acceptability, CHC and health care staff, patients, and community advisory board (CAB) members contributed to LFLS sessions in focus group discussions. Content was pertinent to the cultural, social and religious issues and beliefs influencing EIMC decision making, and included previous Zambian research on this topic [15, 17].

Session 1 focused on the immediate benefits associated with EIMC: Cost effective, faster wound healing, easier to circumcise, less chance of adverse events, easier to clean the penis, reduced urinary tract infections. Masculine cultural norms related to potential pain, sexual performance, and pleasure were addressed during the S&S program; couples learned that potential pain is diminished if the procedure is completed during infancy. Pregnancy heightens awareness of danger and safety for the infant, and the desire to protect the infant. Couples were encouraged to weigh both present and future benefits and risks for the male child, utilizing a cost/benefit approach that incorporates cultural norms regarding the health and wellbeing of all family members. Session 1 included a testimonial from a medical practitioner with EIMC experience, and at the end of the session, couples were invited to return to Session 2 with an influential other, if desired. EIMC brochures were distributed.

Topics included: Why undergo EIMC? What are the benefits? Why now? When is the best time for EIMC? Information: What happens? How long does it take? Is it painful, how is pain controlled? What happens next? How long is healing, cleaning during healing? What happens to the foreskin? What are the risks? Is it safe? Who should be involved in the decision? Any additional questions or concerns about EIMC? The feasibility and acceptability of LFLS was assessed by assessing participants about the utility of each of these topics during their last assessment (see Table 2).

#### LFLS quality assurance

The PI, Co-PIs, consultants, and study staff trained CHC staff in study protocols, including recruitment and intervention procedures. The US and Zambia teams held biweekly calls to review the study progress. Semi-annual visits were made by members of the US team for protocol review, ongoing process monitoring and quality assurance; process evaluation is used to ensure fidelity to the study protocol. All LFLS sessions were recorded; 1) 10% of recordings were randomly reviewed and rated for fidelity by the Project Coordinator, and 2) feedback provided to the counselor within one week of the review; 3) all reviewed recordings would be entered in a spreadsheet detailing completed intervention components that 4) were reviewed by the US Co-PI (Jones); 5) the Project Director and the US PI discussed the reviews to resolve fidelity issues collaboratively. ACASI assessment quality assurance were conducted by US data manager biweekly. Quarterly meetings of CHC and S&S staff were held to encourage team members to review study issues and discuss and resolve difficulties in a timely manner.

### Statistical analyses

Participant characteristics were summarized using descriptive statistics and compared between couples electing EMIC vs. those not circumcising their newborns using chi-square tests. Data were collected from both couple members; where there were high levels of agreement (e.g., on household income or marital status) men’s responses have been reported. As there was no control group in this pilot study, the proportion of couples electing EMIC was compared to an estimated historical rate of 11% [40] as well as hypothetical rates up to 30% using one-sample proportion tests based on previous rates in Zambia [17, 40, 41]. Factors potentially influencing EMIC were examined using a multiple logistic regression model; variables associated with EMIC among either men or women at a liberal *p* value of .2 in bivariate tests were included in the model. Changes in father and other son(s) (i.e., older, non-newborn) VMMC status and plans to undergo circumcision following Spear & Shield were analyzed using mixed effects logistic regression. Time (pre- vs. post-intervenion) was the predictor of interest and random intercepts were estimated for fathers (for father VMMC status) or subjects within couples (for other son(s) VMMC status). Finally, EMIC decision making data were summarized among the couples who elected to have their newborn circumcised. All statistical analyses were conducted using R v.4.2.1.

## Results

### Participant characteristics and EMIC

Participants were *N* = 150 couples delivering a male infant. Demographic characteristics of male and female members of each couple, overall and split by EMIC status, are presented in Table 1. Men were 31 (*SD* = 6) years of age, overall, while women were 26 (*SD* = 5). More men were employed (83%) than their female partners (33%), although rates of higher education (more than primary school) were similar (~70% for both men and women). Most couples (72%) reported a household income of less than 5,000 Zambian Kwacha ($245 USD) per month, which is on parr with the average Zambian household income of $211 USD. Eighty-seven percent of men and 90% of women reported being married, 76% reported living with their spouse or partner, and two-thirds of each gender reported having other children. Eight percent of men and 14% of women reported HIV-positive serostatus. Ninety-one percent of couples received the LFLS and S&S intervention sessions. Importantly, no adverse events were reported by any of the participating couples, for the male partner and all their sons. Participants’ responses to the feasibility and acceptability of LFLS, assessed by assessing participants about the utility of each of the topics covered during sessions, are presented in Table 2.

**Table 1:** Participant characteristics.

	Overall *N* = 150 couples/300 individuals n (%)/mean (sd)	No EMIC *N* = 98 male infants	EMIC *N* = 52 male infants	*p*
Age				
Male partner	30.58 (6.46)	30.64 (6.74)	30.46 (5.94)	.871
Female partner	26.01 (5.33)	26.05 (5.71)	25.92 (4.57)	.888
Full-time Employment				
Male	125 (83.3)	82 (83.7)	43 (82.7)	.999
Female	49 (33.3)	32 (33.3)	17 (33.3)	.999
Household income*				
<5k Kwacha	108 (72.0)	68 (69.4)	40 (76.9)	.501
5k-15k Kwacha	32 (21.3)	22 (22.4)	10 (19.2)	
>15k Kwacha	10 (6.7)	8 (8.2)	2 (3.8)	
Education				
Male				.489
None	14 (9.3)	7 (7.1)	7 (13.5)	
Primary	30 (20.0)	18 (18.4)	12 (23.1)	
Secondary	65 (43.3)	45 (45.9)	20 (38.5)	
Tertiary	41 (27.3)	28 (28.6)	13 (25.0)	
Female				.730
None	15 (10.2)	8 (8.3)	7 (13.7)	
Primary	29 (19.7)	20 (20.8)	9 (17.6)	
Secondary	76 (51.7)	51 (53.1)	25 (49.0)	
Tertiary	27 (18.4)	17 (17.7)	10 (19.6)	
Has other children				
Male	101 (67.3)	63 (64.3)	38 (73.1)	.363
Number	1.77 (1.42)	1.81 (1.48)	1.69 (1.31)	.642
Female	98 (66.7)	61 (63.5)	37 (72.5)	.358
Number	1.73 (1.47)	1.59 (1.43)	1.98 (1.53)	.129
Married*	131 (87.3)	83 (84.7)	48 (92.3)	.282
Lives with partner*	114 (76.0)	72 (73.5)	42 (80.8)	.426
HIV status				
Male				.806
Negative	134 (89.3)	88 (89.8)	46 (88.5)	
Positive	12 (8.0)	8 (8.2)	4 (7.7)	
Don’t know	4 (2.7)	2 (2.0)	2 (3.8)	
Female				.147
Negative	125 (85.0)	83 (86.5)	42 (82.4)	
Positive	20 (13.6)	13 (13.5)	7 (13.7)	
Don’t know	2 (1.4)	0 (0.0)	2 (3.9)	
Circumcised (males)	88 (58.7)	53 (54.1)	35 (67.3)	.164
Has plans for MC (if uncircumcised)	49 (79.0)	36 (80.0)	13 (76.5)	.999
Has sons who are circumcised				
Male	32 (21.3)	16 (16.3)	16 (30.8)	.065
Female	29 (19.7)	16 (16.7)	13 (25.5)	.288
Intends to circumcise future sons				
Male	100 (87.7)	67 (87.0)	33 (89.2)	.979
Female	100 (86.2)	64 (85.3)	36 (87.8)	.930
Received Spear & Shield intervention	137 (91.3)	91 (92.9)	46 (88.5)	.545

*For characteristics that were very similar within couples, we report men’s data

**Table 2:** Feasibility and acceptability of LFLS.

Variable	N (%)
Duration of Sessions	
Just right	505 (88.9%)
Too long	32 (5.6%)
Too short	31 (5.5%)
Use/Utility of Sessions	
No	13 (2.3%)
Yes	555 (97.%)
Use/Utility of HIV/STI Discussions	
No	11 (1.9%)
Yes	553 (97.4%)
Use/Utility of Infant Circumcision Procedure Discussions	
No	15 (2.6%)
Yes	553 (97.4%)
Use/Utility of Foreskin Disposal Discussions	
No	33 (5.8%)
Yes	535 (94.2%)
Use/Utility of Healing and Recovery Discussions	
No	24 (4.2%)
Yes	544 (95.8%)
Use/Utility of Post Circumcision Pain Discussions	
No	42 (7.4%)
Yes	526 (92.6%)
Use/Utility of Circumcision and Fertility Discussions	
No	21 (3.7%)
Yes	547 (96.3%)
Use/Utility of Circumcision and Sexual Performance Discussions	
No	21 (3.7%)
Yes	547 (96.3%)

### EMIC rates

Fifty-two of 150 couples elected to have their newborn son circumcised (35%). Although there was no control group in this pilot/feasibility study, the study EMIC rate of 35% was significantly higher than the estimated EMIC rate of 11% previously observed in Zambia (*p* < .001; [40]). The study EIMC rate remains higher than comparison hypothetical EIMC rates up to 30% (*p* = .034), which is substantially higher than even optimistic estimates of historical rates in Zambia. No baseline demographic or other participant characteristics were significantly predictive of EIMC in this study.

### Father and family VMMC status

At baseline, fifty-nine percent of fathers reported being circumcised (*n* = 88), and among those who were not, 49/62 (79%) reported having plans to be circumcised in the future. There was no evidence supporting changes in father self-reported MC status or plans to become circumcised following the Spear & Shield intervention in the current study, though previous studies of S&S found the intervention to influence adult men to undergo VMMC (4). Twenty percent of the 150 couples had other sons who were circumcised, and 88% of men and 86% of women said that they intended to circumcise future sons. Following the LFLS intervention, couples were more likely to report having a male son (other than the newborn) circumcised; odds (non-significantly) increased by 31% at post-intervention [20% to 23%; OR = 1.31, 95% CI 0.79 to 2.18, *p* = .30] and 90% at two-weeks following birth, relative to baseline [20% to 28%; OR = 1.90, 95% CI 1.15 to 3.15, *p* = .013].

### Drivers of decision-making

Post-EIMC decision-making data were available for at least one member of 47/52 couples who elected to have their newborn son circumcised. Eighty percent of men and 90% of women reported that EIMC was a shared decision between couple members, while 15% of men and 7% of women indicated that it was their decision alone. Similarly, 82% of men and 93% of women reported that both couple members were present for their newborn’s circumcision procedure. One man and three women said that the EIMC decision caused a serious disagreement with their partner. Women (50%) were more likely than men (24%) to have consulted their mother prior to deciding to circumcise, but there was little difference between men and women in terms of consulting their fathers (26% and 32%, respectively). In addition to parents, couples were likely to have discussed circumcision with friends (48% of women and 37% of men). Proportions of participants consulting grandmothers, sisters, uncles, or aunts were generally in the 15–30% range. Across all persons consulted, participants were advised that circumcision was a good idea 80–90% of the time, with grandmothers least likely to be supportive (67% supported EIMC).

## Discussion

This feasibility study assessed the potential for the LFLS intervention to promote EIMC uptake above EIMC rates previously observed in Zambia, building on the successful implementation and dissemination of the S&S intervention across a series of studies [42, 43]. In addition, we assessed changes in fathers’ and other sons’ (e.g., adolescents) VMMC status. Overall, the study achieved an EIMC rate of 35%. Compared to a previously observed EIMC rate in Zambia (11%) [40], this study rate was significantly higher, and remained significantly higher than comparison hypothetical EIMC rates up to 30%. Further, other than their newborn, couples also reported having a male son circumcised following the intervention. Specifically, odds of VMMC among older sons non-significantly increased by 31% at post-intervention and by 90% at two-weeks following birth, relative to baseline. Most participants found the topics covered during sessions to be acceptable, and the duration of the sessions was deemed to be feasible.

Findings from this study were promising and suggest that LFLS was successful in increasing EIMC rates as well as older sons’ VMMC status. About ~361,000 male babies are born each year in Zambia, most of whom would be candidates for EIMC, and substantial research has already been conducted on factors related to parents’ decisions to circumcise their sons [15, 44–47]. However, growing evidence suggests that parents are willing to circumcise their sons, and we are unaware of any major evidence-based effort in sub-Saharan Africa to stimulate prospective parents in the context of male involvement antenatal programs to simultaneously consider EIMC and VMMC for older sons and fathers. Indeed, rates of VMMC were higher among other sons, other than newborns, than rates of EIMC. These findings suggest the LFLS intervention may have been more effective in increasing uptake of MC by older sons than newborns.

Despite the promising findings in favor of EIMC rates and other sons’ VMMC rates from this study, fathers’ VMMC status were not affected by the intervention. This finding may be related to the emphasis of the intervention on the benefits of, and lower risks associated with EIMC compared to adults’ VMMC. Indeed, research suggests that the neonatal period is the ideal time for male circumcision to be performed. In a 2012 review, the American Academy of Pediatrics concluded that health benefits of newborn circumcision not only outweigh the risks, but also justify being accessible to all families that choose this option [48]. Long-term benefits include the prevention of HPV transmission (as a precursor to cervical cancer among adult women) as well as a reduction in risk for UTIs, HIV, and penile cancer [50]. A more immediate benefit includes an improvement in hygiene [15]. In addition to these benefits, significant complications of this procedure are rare. Further, EIMC is safer, less costly, and has shorter healing times compared to MC among adolescent and adult men, when more physical movement and sexual activity is involved, which increases the risk for complications and healing time [12–15]. These benefits are enhanced by the fact that any risk for potential surgical complications during the neonatal period are minimized, and any emerging surgical complications are more amenable to intervention in the neonatal period, compared with adults [49]. Most importantly, the protective effects of EIMC are conferred immediately and these protective effects starting at an early age may accumulate over a lifetime [15]. Studies that follow families over time are needed to assess these long-term benefits of EIMC in Zambia. In addition, future intervention studies using LFLS may continue to emphasize the benefits of VMMC *despite* its higher risks compared to EIMC.

We examined demographic and other participant characteristics in relation to EIMC. No baseline demographic or other participant characteristics were significantly predictive of EIMC. Previous studies have assessed parents’ attitudes towards EIMC [47]. Noted associations with EIMC include higher socioeconomic status, fathers’ older age, religious beliefs, perceived benefits, and education [15, 44–47]. Parents in this study were followed over time, whereas previous studies were cross-sectional in nature, which may explain these different results; this represents a strength of our study. Previous studies have indicated that men’s decision-making process to undergo VMMC may be characterized by “stages of change” that are not static [50]. Couples decision-making may be similar, such that longitudinal studies may provide different information about how couples makes decisions about their sons’ circumcision status. The lack of baseline predictors of EIMC, however, may also be influenced by the relatively small sample size of the study. Larger samples sizes may still be needed to not only assess EIMC predictors in the context of an intervention, but also to assess, for example, potential cultural mediators of EIMC and family VMMC status.

This study makes an important contribution to the literature on EIMC, but it is not without limitations. First, the LFLS intervention was conducted in four urban community health centers in Lusaka District, Zambia. As our previous studies have shown, the implementation and dissemination of S&S carried unique challenges at each province during implementation [20, 42, 51]. Therefore, the results from this study may not generalize to other provinces outside of Lusaka or other countries with high rates of HIV. Second, though we began with 702 participants amounting to 351 couples, the final analytic sample with male infants was only 150 couples, a relatively small sample size. Future research recruiting large sample sizes of couples across Zambia are needed.

This pilot study found the Like Father Like Son intervention to be feasible, acceptable, and effective in doubling the rate of EIMC in comparison with a previous Zambian study. Future research should consider a family-centric approach to promotion of male circumcision for infants and adolescents. LFLS during the perinatal period may be effective in promoting both VMMC in sons and EIMC in neonates which may create a novel leverage point for promotion of this HIV prevention strategy.

## Supporting information

S1 File (PDF)

S2 File (CSV)
