# Supplementary material for: Increasing early infant male circumcision uptake in Zambia: Like father like son
Source: PLoS One. 2023 Aug 10;18(8):e0289819. doi: 10.1371/journal.pone.0289819 (PMC10414584; doi:10.1371/journal.pone.0289819)
Supplement: S1 File — (PDF) [file pone.0289819.s001.pdf]

UNIVERSITY
OF MIAMI

University of Miami
Human Subject Research Office
Gables One Tower
1320 S. Dixie Highway, #650
Coral Gables, FL 33146

Ph.: 305-243-3195
Fax: 305-243-3328
www.hsro.med.miami.edu

APPROVAL

February 16, 2023

Stephen Weiss
sweiss2@miami.edu

On 2/16/2023, the IRB reviewed and approved the following submission:

Type of Review:	Modification / Update
Title of Study:	Increasing early infant male circumcision uptake in Zambia: Like Father Like Son
Investigator:	Stephen Weiss
IRB ID:	MOD00003594
Funding:	Name: National Institute of Mental Health (NIMH), Funding Source ID: R01MH112111
IND, IDE, or HDE:	None
Documents Reviewed:	• HRP-503 - PROTOCOL Like Father Like Son 1.2 CT.pdf, Category: IRB Protocol;
Reconsenting Instructions:	- N/A: Revisions were not made to ICF
Reviewing IRB:	

Conditions of Approval - Please Read

1. In conducting this study, you are required to follow the requirements listed in the Investigator Manual (HRP-103). The PI is attesting to take full responsibility for the conduct of the study with any approval.

2. This approval is limited to the documents and items in the submission referenced above.

3. Approval from the IRB is required before making any modifications to the research unless the modification is necessary to prevent a subject from experiencing imminent harm.

4. You must submit Reports of New Information as required in the UM Investigator Manual HRP- 103, Chapter 8.

5. You must obtain IRB approval of translated documents before using them.

6. **If you plan to conduct this study at JHS, before conducting any research procedures at JHS, you must:**
    - **Indicate this intention on the Initial Review Smart Form;**
    - **Submit an application to JHS;**
    - **Receive an approval letter from the JHS Clinical Research Review Committee (CRRC); and**
    - **Submission of a participant's signed consent form(s) to the JHS Clinical Research Office is required within two business days of enrollment.**

If you have any questions regarding this process, please contact the JHS Office of Research at 305-585-7226.

7. Approval of this study does not expire. However, you must submit a Continuing Review Report when you are ready to close this study. As a reminder, you should close studies when:
    - You are no longer interacting or intervening with human subjects to collect data about them; and
    - You are no longer accessing private identifiable information.

8. If the study requires pathology research services, such as the collection of biospecimen, request for archival tissue, or the processing of samples, prior approval from the Pathology Ancillary Review Committee is needed.

    If you have any questions regarding this process, please email the Department of Pathology & Laboratory Medicine: DPLMresearch@miami.edu.

Should you have any questions, please contact: Yaslaime Fraga, IRB Regulatory Analyst, (phone: +1 (305) 2439925; email: y.fraga1@miami.edu).
